# Comparison of competency priorities between UK occupational physicians and occupational health nurses

**DOI:** 10.1136/oemed-2016-104049

**Published:** 2017-02-23

**Authors:** Drushca Lalloo, Evangelia Demou, Marisa Stevenson, Mairi Gaffney, Ewan Beaton Macdonald

**Affiliations:** 1 Healthy Working Lives Group, Institute of Health and Wellbeing, College of Medical, Veterinary and Life Sciences, University of Glasgow, Glasgow, UK; 2 MRC/CSO Social and Public Health Sciences Unit, Institute of Health and Wellbeing, College of Medical, Veterinary and Life Sciences, University of Glasgow, Glasgow, UK; 3 Paisley Campus, University of the West of Scotland, Glasgow, UK

**Keywords:** competencies, occupational physician, occupational health nurse, training, Delphi study

## Abstract

**Objectives:**

The competencies required of occupational physicians (OPs) and occupational health nurses (OHNs) separately have been studied in various countries but little research has made direct comparisons between these two key occupational health (OH) professional groups. The aim of this study was to compare current competency priorities between UK OPs and OHNs.

**Methods:**

A modified Delphi study conducted among professional organisations and networks of UK OPs and OHNs. This formed part of a larger Delphi, including international OPs. It was undertaken in two rounds (round 1—‘rating’, round 2—‘ranking’), using a questionnaire based on available OH competency guidance, the literature, expert panel reviews and conference discussions.

**Results:**

In each round (rating/ranking), 57/49 and 48/54 responses were received for OPs and OHNs respectively. The principle domain (PD) competency ranks were very highly correlated (Spearman’s r=0.972) with the same PDs featuring in the top four and bottom three positions. OPs and OHNs ranked identically for the top two PDs (good clinical care and general principles of assessment and management of occupational hazards to health). Research methods was ranked lowest by both groups.

**Conclusions:**

This study has observed a high level of agreement among UK OPs and OHNs on current competency priorities. The ‘clinically focused’ competency priorities likely reflect that although OH practice will broaden in response to various factors, traditional ‘core’ OH activities will still be required. These mutually identified priorities can serve to strengthen collaboration between these groups, develop joint education/training programmes and identify common professional development opportunities.

**Key messages**

**What this paper adds**

- Occupational physician (OP) and occupational health nurse (OHN) competencies are complementary and separately have been studied in various countries, but this is the first study to compare competency priorities between UK OPs and OHNs.
- This study reports a very high level of agreement on the identified competencies, with the same principle domains featuring in the top four and bottom three ranks.
- These mutually identified priorities can strengthen collaboration between these groups and identify common professional development/education opportunities for their respective governing bodies.

## Introduction

Occupational health (OH) is a well-established discipline in the UK. Persisting practice challenges include improving workforce access to such services, matched with a shortage of OH professionals, notably physicians.^1^ Despite these challenges, OH is rapidly evolving with advances in technology, workplace/workforce changes and socioeconomic factors. Modern UK OH teams are often multidisciplinary with a range of practitioners including physicians, nurses, hygienists, technicians, physiotherapists, psychologists and ergonomists. It has been advocated that, given restricted workforce access to OH services and shortfalls in OH resources, OH professional’s skills and competencies should be examined to determine who can do what and to ensure the appropriate use of resources.^1^ UK occupational health nurse (OHN) roles have expanded over time with the first OHN consultant post in 2002, many OHNs leading services and a UK Faculty of Occupational Health Nursing (FOHN) being established (http://www.fohn.org.uk/). By nature, there is a degree of overlap in clinical activity between OH professionals but service provision models have also developed where certain tasks traditionally only performed by doctors (eg, management referrals) are now being undertaken by nurses. Conversely, certain nursing tasks (eg, routine health surveillance) are now being undertaken by technicians. This may be driven by financial reasons or may illustrate the Abbott model ‘interdependency’ concept, where professions working together overlap as they compete for jurisdiction, as new knowledge and skills are acquired and as particular service demands increase or decrease with the changing workplace.^2^ In any multidisciplinary OH team, a close working relationship between occupational physicians (OPs) and OHNs is required, and their competencies understood. This is important for clinical governance and identifying ongoing training requirements. OP and OHN competency requirements separately have been studied in various countries,^3–9^ but little research has made direct comparisons between these two professional groups.^7^ A global survey comparing work jurisdiction of OH professionals demonstrated substantial differences for OPs and OHNs in required competency sets, although this was undertaken over a decade ago.^7^

OH practice evolution with expanding and overlapping OP and OHN roles make it imperative that up-to-date competencies reflective of practice are established. The aim of this study was to compare current competency priorities between UK OPs and OHNs. A literature review has not identified any study (UK or internationally) specifically assessing competency priorities between these two groups.

## Methods

A modified Delphi study was conducted among professional organisations and networks of UK OPs and OHNs. This formed part of a larger international Delphi and a comprehensive description of the methodology used, the international OP and individual UK OHN results are presented elsewhere.^8 9^ It was conducted in two rounds using a developed questionnaire based on training/competency guidance for OH practitioners, the literature, expert panel reviews and conference discussions. The initial questionnaire comprised 12 principal domains (PDs), each with detailed subsection items, covering different practice areas within that domain. In round 1, respondents were asked to ‘rate’ the relative importance of the items.

For round 2, a revised questionnaire was produced retaining the same 12 PDs but including new subsection items suggested by first-round respondents from open-ended questions.^8^ Respondents were asked to ‘rank’ the PDs and the domain subsections.

Both questionnaires were distributed using a SmartSurvey link and circulated by the same UK OP and OHN professional organisations and networks. OPs and OHNs receiving the link were invited to participate irrespective of whether they had taken part in the previous round. The University of Glasgow, College of Medical, Veterinary and Life Sciences Ethics Committee provided ethics approval (200130150).

## Results

Round 1 (Rating): For OPs and OHNs, respectively, 57 and 48 responses were received with OPs predominantly male (61%) and OHNs female (96%). In both groups, the majority were over 45 years with a mean 20 and 18 years of experience, respectively. Industry, other and healthcare services were the main practice areas, with some crossover.

All 12 PDs were considered important with 88% and above ‘yes’ responses in both groups. A small number in each perceived some competencies as not relevant to their practice. 12% of both OPs and OHNs considered research methods not relevant.

Round 2 (Ranking): For OPs and OHNs, respectively, 49 and 54 responses were received with OPs predominantly male (69%) and OHNs female (94%). Similar to round 1, the majority of OPs and OHNs were over 45 with a mean 23 and 19 years of experience, respectively. Industry and healthcare services were the main practice areas, again with some crossover. 33% of round 2 participants had participated in round 1. A statistically significant difference between respondents of both rounds was demonstrated only for job title.

**Figure 1:**
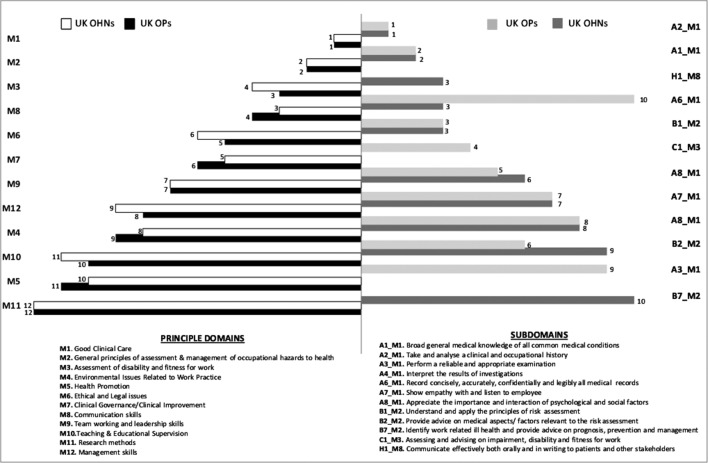
Principal domain and top 10 subsection rankings overall for occupational physicians (OPs) and occupational health nurses (OHNs).

The PD and top 10 subsection rankings overall are presented in figure 1. The same PDs featured within the top four and bottom three positions for both groups with all PDs only ranking differently by one rank in either direction overall. Both groups ranked identically on importance for the top two PDs, that is, good clinical care and general principles of assessment and management of occupational hazards to health. Research methods were ranked least important by both groups.

Testing for PD ranking differences within our sample, the competency ranks are highly correlated (Spearman’s r=0.972), indicating no difference between the OP and OHN responses.

As the PDs represent broad categories that may reduce the differences identified between the groups, we weighted and ranked the subsection items within each PD and overall to detect finer group differences. The top ranked subsection within each PD was exactly matched in all except one PD (law and ethics) between the groups. The top and bottom two ranked subsection items across all PDs were also identical for both groups. The top two items (see figure 1) both pertained to good clinical care reflecting ‘core’ OH activities. The bottom two subsections related to research including ‘carrying out basic statistical analyses’ and ‘presenting research reports’. While this demonstrates high correlation even at subsection level, the few outliers observed among OPs pertained to assessment and advisory functions while for OHNs they related to administrative tasks and communication.

## Discussion

This study has compared current competency priorities for OH practice between UK OPs and OHNs. It has identified a very high level of agreement among respondents on the identified competencies both by broad category and specific descriptive subsection level. This agreement is a likely reflection of OH practice evolution, including the Abbott model ‘interdependency’ concept, describing overlap in tasks/roles of professions working together.^2^ It may also reflect modern multidisciplinary OH practice and closer team working.

Examining the competency priorities themselves, respondents maintain more traditional ‘clinically focused’ views of required competencies. This likely reflects that although the scope of OH practice will broaden in response to various factors, basic ‘core’ OH activities still dominate day-to-day clinical practice.

To our knowledge, this is the first study to compare perceived competency priorities between these two key OH professional groups. Its strengths are that consensus has been derived from respondents working across various sectors and that, in a rapidly evolving discipline, it has captured real-time developments reflective of current practice.

A study limitation is the low response rate, making it difficult to establish the representativeness of these results across all UK OPs and OHNs. Similar challenges have been encountered in other online surveys and competency studies.^7–10^

Our findings show some correlation with previous individual OP and OHN studies,^4–6^ with ‘clinically focused’ competencies a high priority and research a lower priority, but none of these made comparisons between the professional groups. Substantial differences in required competency sets for OPs and OHNs were observed in a global survey on work jurisdiction of OH professionals undertaken over a decade ago.^7^ Our contrasting, more highly correlated findings may be explained by OH practice evolution already described and service provision/multidisciplinary models currently in place. Based on these findings and if increasing alignment with physician competencies is envisioned, current UK OHN training curricula^11^ need to be strengthened, particularly on the clinical assessment process and the elements of good clinical care defined in this study.

These mutually identified priorities can assist in the development of common core training within multidisciplinary OH teams where overlap exists and inform effective service delivery models. Joint education will have the added advantage of expanding appreciation of fellow professional’s roles and promoting mutual respect towards each other's competencies.^12^ In the UK, this study is timely with the recent establishment of the FOHN, identifying areas of common ground for education/training and professional development, for potential collaboration with the Faculty of Occupational Medicine. Revalidation is also a driver to pool common training and professional development resources. The relevance of these findings extends beyond the UK to other countries such as the USA and Australia, where multidisciplinary OH practice is developed, and to those in which it inevitably will develop. Follow-on research opportunities could include in-depth exploration of the drivers for expanding and decreasing OH functions and evaluating different OH service delivery models.
